# Clotrimazole as a new frontier: Drug repurposing and its efficacy in cancer therapy

**DOI:** 10.1016/j.cpt.2025.03.004

**Published:** 2025-03-26

**Authors:** Shubham C. Karpe, Manjula Kiran, Sukhen Majhi, Jaipal Meena, Rajesh Kumar, Harish Chander, Anupkumar R. Anvikar, Harit Kasana

**Affiliations:** a Vaccine and Antisera Laboratory, National Institute of Biologicals, Plot No. A-32, Sector-62, Noida, Uttar Pradesh-201309, India; b Department of Pharmaceutical Sciences, School of Pharmaceutical Sciences, Lovely Professional University, Phagwara, Punjab-144411, India

**Keywords:** Clotrimazole, Antineoplastic agents, Neoplasms, Antifungal agents, Immunomodulation

## Abstract

Cancer, ranging from early stages to metastatic spread, is one of the leading causes of death globally. Current treatment options, including chemotherapy, radiotherapy, and targeted drugs, have limitations substantial adverse effects, the development of drug resistance, and high cost. To address these challenges, numerous studies have focused on repurposing existing drugs for anticancer therapy, with clotrimazole (CLZ) emerging as a promising candidate due to its notable anticancer activity. CLZ was first developed as an antifungal agent. Recently, significant anticancer effects have been observed making it a suitable candidate for drug repurposing. Compared with other azole-based antifungals, CLZ has shown distinct therapeutic effects on cancer cells via several pathways. Its ability to disrupt glycolysis by inhibiting phosphofructokinase (PFK) and hexokinase (HK) distinguishes it from other azoles. Furthermore, CLZ obstructs calcium homeostasis and critical survival pathways, such as extracellular signal-regulated kinase (*ERK*)*-p65*, phosphatidylinositol 3-kinase (*PI3K*), and mitochondrial apoptotic pathways, inhibiting tumor growth, inducing apoptosis, and attenuating metastasis. This review summarizes the potential of CLZ repurposing for cancer therapy, emphasizing its well-established safety profile and cost-effectiveness while addressing unmet clinical needs in current cancer treatment. It briefly examines *in vitro* and *in vivo* assessments to understand the mechanisms and effects of CLZ on various cancer types. Furthermore, novel strategies such as nanoformulations and combination therapies with existing chemotherapeutic drugs have been highlighted to improve therapeutic outcomes. Preclinical studies have provided promising evidence for the efficacy of CLZ in different cancers, showing tumor regression and improved responses to conventional chemotherapy or targeted therapies. Given its evident preclinical results and diverse mechanisms of action, CLZ may be considered an antineoplastic agent. Further clinical research is required to fully elucidate its anticancer potential, potentially positing it as a valuable addition to currently available cancer treatments.

## Highlights

• Clotrimazole (CLZ) is an antifungal which has the potential to be repurposed for anticancer therapy.
• CLZ disrupts the tumor cell cycle and DNA synthesis.
• CLZ induces apoptosis via extracellular signal-regulated kinase -p65, phosphoinositide 3-kinase, calcium ions (Ca^2+^), potassium ions (K^+^) channels, and mitochondria regulating cell death pathways.
• CLZ inhibits phosphofructokinase (PFK) and hexokinase (HK), and modulates apoptotic proteins, affecting cell survival.
• CLZ shows promise in combination therapies for overcoming anticancer drug resistance.

## Graphical abstract

**Figure:**
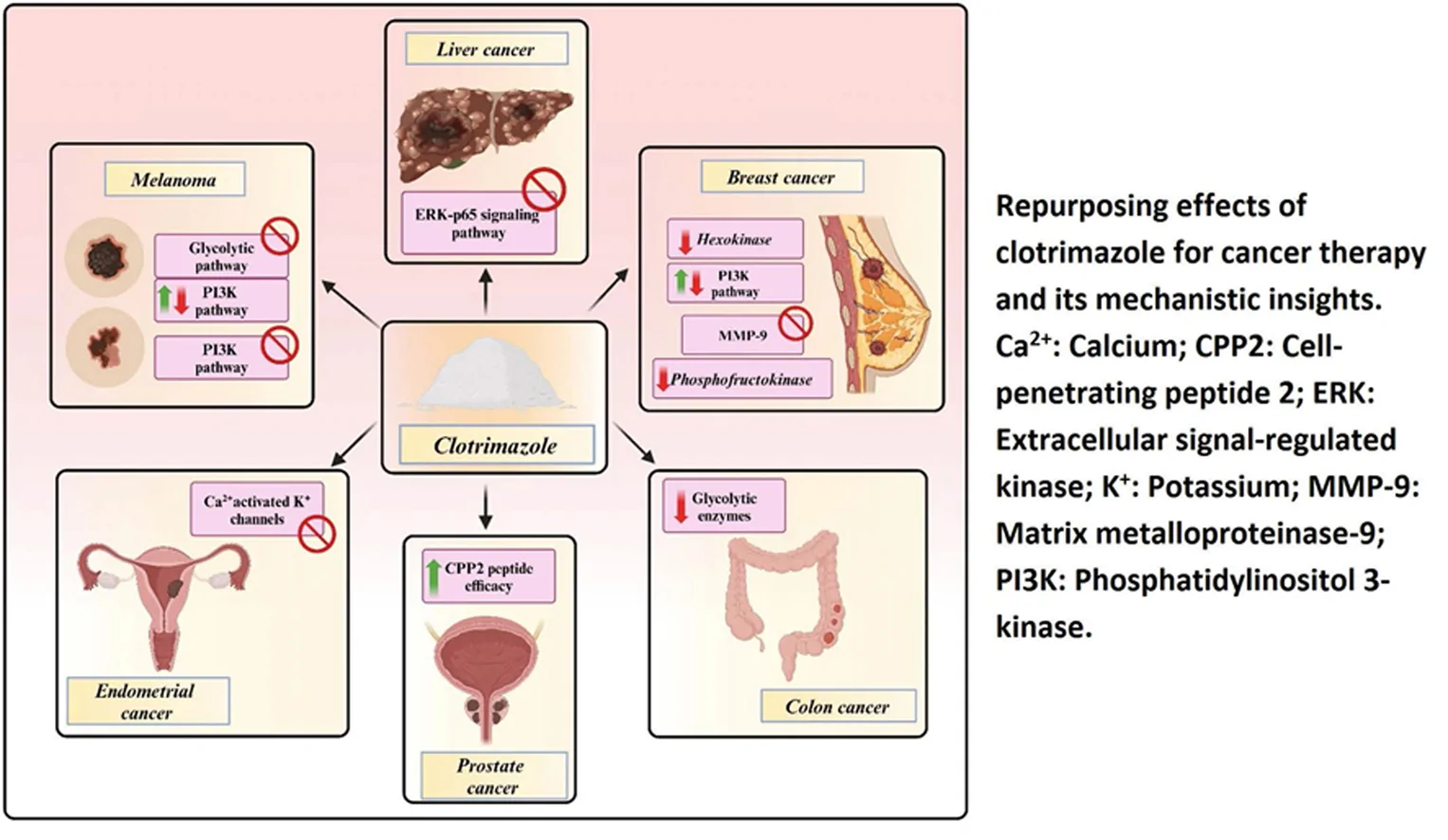

## Introduction

Cancer is the second leading cause of mortality globally, responsible for approximately one in every six deaths. In 2020, there were approximately 19.3 million new cases of cancer worldwide (18.1 million excluding non-melanoma skin cancer) and >10 million cancer-related deaths (9.9 million excluding non-melanoma skin cancer), with 70% being in low- and middle-income countries.^1^ Drug repurposing refers to the reutilization of existing or investigational drugs for new therapeutic purposes. This approach has gained attention because of its potential to save time and money in drug development, as well as reduce the risks associated with clinical development. Repurposing antifungal drugs for cancer therapy has attracted unprecedented attention in both preclinical and clinical research because of specific advantages such as safety, high-cost effectiveness, and time-saving compared with cancer drug discovery.^2^^,^^3^ Recently, various non-oncology drugs have been discovered for their anticancer activity based on drug repurposing strategies, among which antifungal agents have attracted considerable attention.^4^

Clotrimazole (CLZ) is a synthetic azole derivative with antifungal activity. In late 1972, Karl Heinz Buchel synthesized CLZ, which was commercialized in Germany as Canester.^5^ Owing to its effect on sickle cell anemia, malaria, and cancer, it is on the World Health Organization's essential drug list. Researchers have demonstrated its anticancer efficacy against lung, colorectal, breast, and endometrial cancer cell lines. CLZ is highly effective against cancer cells and is not harmful to healthy cells.^6^ *In vitro* and *in vivo* tests on human melanoma and glioblastoma cells have shown that CLZ inhibits cancer cell proliferation.^7^ This is because most tumors depend on hyperactivated phosphatidylinositol 3-kinase (*PI3K*) and the glycolysis pathway to survive, both of which are affected by CLZ.^8^^,^^9^ In this review, we aim to describe the various cancer types that CLZ has been shown to inhibit, as well as the molecular targets and signaling pathways linked to these effects. The chemical, pharmacological, and pharmacokinetic aspects of CLZ are also emphasized. In addition to the future possibilities for its usefulness in cancer treatment, combination treatments and novel drug delivery techniques to boost its anticancer potential have also been explored.^10^ Hence, this review explores the potential of repurposing CLZ in cancer, and its well-established safety profile and cost-effectiveness, to highlight current treatment gaps.

### Chemistry and pharmacology of clotrimazole

**Figure 1:**
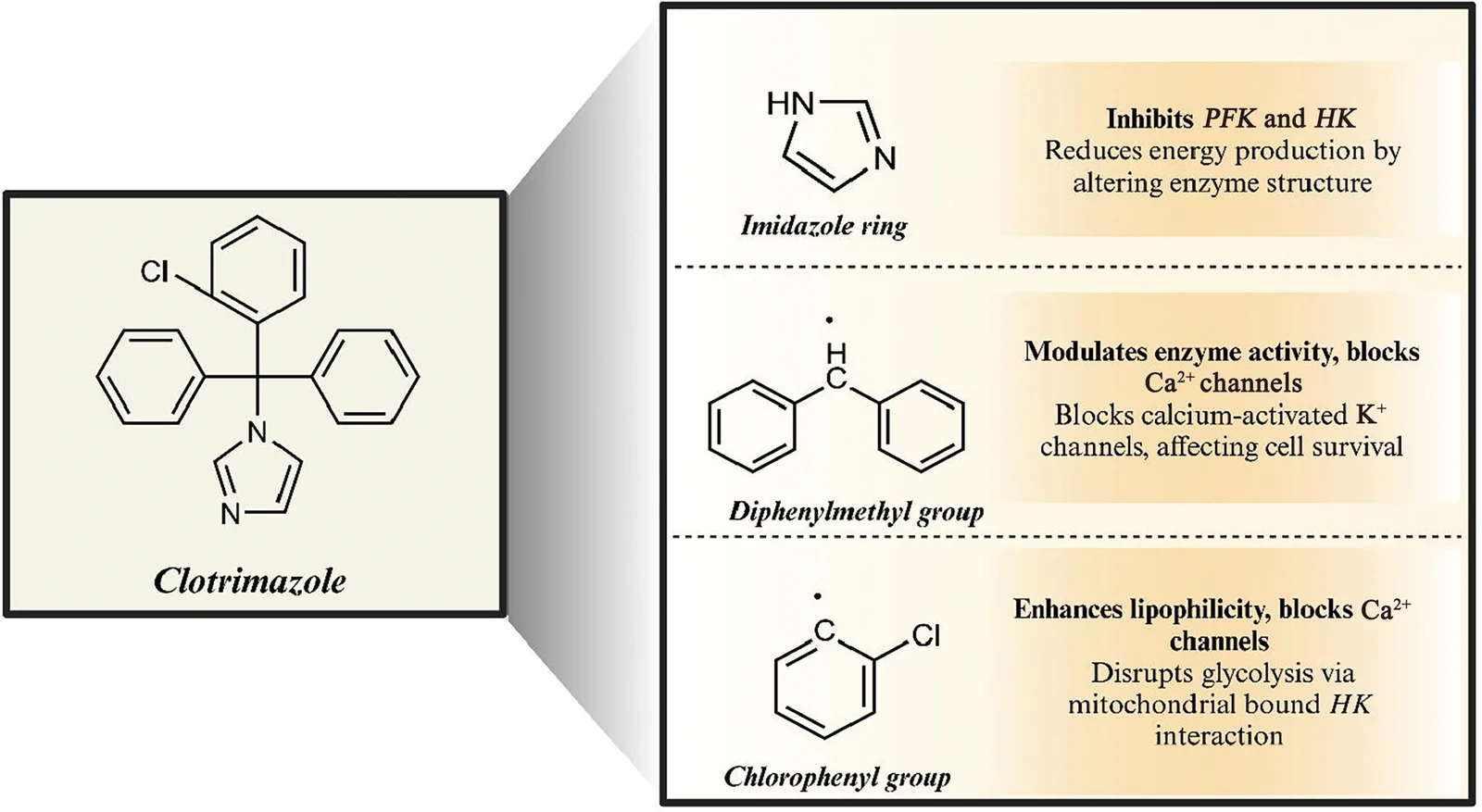
Chemical structure of clotrimazole with details about the groups responsible for its anticancer activity. Ca^2+^: Calcium; HK: Hexokinase; K^+^: Potassium; PFK: Phosphofructokinase.

CLZ is a member of the imidazole-derived antifungal agents. The chlorophenyl moiety contributes to the overall structure of this compound, as shown in Figure 1, and is composed of a 1H-imidazole ring joined to a diphenylmethyl group. Its molar mass is approximately 344.83 g/mol, and its chemical formula is C_22_H_17_ClN_2_.^6^^,^^11^ CLZ possesses distinctive characteristics and pharmacological actions owing to its distinct atomic configuration. Two nitrogen atoms are found in the heterocyclic aromatic imidazole ring of CLZ.^12^ Because of its interaction with fungal cytochrome P450 (CYP450) enzymes,^13^ this ring system inhibits the formation of ergosterol, an essential component of fungal cell membranes, which gives CLZ its antifungal function.^14^ When topically used, CLZ's chlorinated phenyl ring increases its lipophilicity and facilitates its absorption into biological membranes, such as the stratum corneum of the skin. CLZ's antifungal mechanism also involves the inhibition of lanosterol 14α-demethylase, which is a vital enzyme responsible for ergosterol production.^15^ By affecting these cellular mechanisms, CLZ damages the integrity of fungal cell membranes, resulting in increased permeability and cell death.^16^

CLZ is predominantly administered topically due to its poor systemic bioavailability. Different formulations such as oral troches, ointments, lotions, and powders provide flexibility in treating many types of fungal infections.^17^ Local absorption of the drug occurs in tissues, followed by hepatic metabolism and excretion of its metabolites in feces. CLZ is indicated for the treatment of dermatophytic infections, including ringworm, athlete's foot, candidiasis, and other fungal skin diseases. It has also been used to treat otomycosis and vaginal yeast infections. Some patients report localized, moderate adverse effects, such as skin irritation and itching. Although systemic drug interactions are rare when using CLZ, monitoring, and expert advice are important to ensure proper and secure administration.^18^ CLZ is soluble in various solvents, including acetone, chloroform, and ethyl acetate; however, it has limited solubility in water, toluene, benzene, ether, and a few other liquids.^19^

In addition to its well-established antifungal capabilities, CLZ has shown encouraging pharmacological benefits in cancer treatment by targeting cellular pathways involved in tumor development and progression. One of the methods CLZ uses to fight cancer is by interfering with the metabolic processes that cancer cells rely on to stay alive. These processes include glycolysis and oxidative phosphorylation.^20^ Furthermore, it causes cancer cells to experience increased oxidative stress and cell death by blocking calcium (Ca^2+^)-dependent potassium (K^+^) channels and mitochondrial activity. Hence, CLZ may find new applications in cancer treatment, especially when coupled with modern drug delivery technologies to increase its bioavailability and tumor-specific targeting. Improving CLZ formulations and determining their therapeutic use in cancer treatment requires further study.^20^

## Pharmacokinetics of clotrimazole

Although CLZ's main application is as an antifungal, its pharmacokinetic profile may be very different for cancer treatment. The pharmacokinetics of CLZ for cancer treatment may be influenced by several factors. First, to achieve systemic exposure, the route of administration may need to be changed from topical to oral or intravenous. Oral administration of CLZ leads to absorption in the gastrointestinal (GI) tract, achieving higher systemic concentrations compared with topical application.^21^ The distribution of CLZ in cancer patients may differ from that in individuals with fungal infections, potentially affecting the amount of the drug that reaches tumor tissues. Hepatic enzymes can break down CLZ when used as an antifungal agent; however, in patients with cancer, changes in enzyme activity caused by illness or concurrent drugs may affect CLZ metabolism. With respect to pharmacokinetic adaptation, increasing systemic administration, increasing solubility and absorption, improving stability, enabling controlled release, and optimizing metabolism as well as clearance are essential. Third, CLZ and its byproducts may be eliminated differently in patients with cancer based on hepatic or renal function, among other considerations. The systemic absorption of CLZ is decreased due to first-pass metabolism in the liver. The plasma levels of CLZ stay below 0.01 μg/mL when given vaginally, and an estimated absorption rate of 3–10% is achieved.^22^ To address these issues, methods such as integrating β-cyclodextrin or nanoparticles demonstrated a boost in CLZ plasma concentrations and oral dissolution rates, hence improving its bioavailability.^23^ Moreover, the integration of CLZ with specific drugs may further boost its bioavailability. Vasquez et al.^24^ found that the oral bioavailability of tacrolimus improved by 250%, whereas its clearance was reduced by 60% when administered along with CLZ. Similarly, CLZ decreased the bioavailability of midazolam by lowering its oral clearance.^25^ To maximize CLZ's dosage schedule, ensure efficacy, and reduce toxicity, a detailed analysis of its pharmacokinetic profile in the context of cancer treatment is necessary.^21^^,^^26^^,^^27^

Recent advances in drug delivery technologies offer new options for modifying CLZ's pharmacokinetic features to boost its therapeutic potential against cancer. Novel drug delivery systems, including polymeric micelles, self-emulsifying drug delivery systems (SEDDSs), and lipid-based nanoformulations, have been developed to prolong systemic circulation time, enhance stability, and improve solubility.^20^ The encapsulation of CLZ into dendrimers or liposomes may enable targeted tumor delivery while decreasing off-target effects. Moreover, prodrug methods and structural changes in CLZ may aid in avoiding metabolic degradation, thereby enhancing plasma half-life and drug retention. Further studies on these methodologies may provide a more efficient method of employing CLZ as a repurposed anticancer drug, improving its pharmacokinetics for systemic delivery, while lowering toxicity and boosting therapeutic effects.^28^

## Anticancer activity of clotrimazole in specific cancer types

Tumor cells utilize glucose as their main energy source; hence, glucose metabolism has been targeted in the recent development of novel drug delivery systems and treatments.^29^, ^30^, ^31^, ^32^ Cancer has the special feature of high fermentative glycolytic flow, even in the presence of a large supply of oxygen, known as the “Warburg effect.”^33^, ^34^, ^35^ Tumors create high levels of lactate because of the Warburg effect, which has many benefits for their invasive and proliferative abilities.^32^^,^^33^ The primary regulating glycolytic enzyme, PFK, has been linked to increased lactate generation.^33^^,^^36^ The rate of lactate generation and overall glycolytic flux are both linked to PFK activity.^37^ Previous studies have shown that CLZ directly suppresses PFK activity by altering its quaternary structure and substrate affinity^8^; however, it is unclear whether this inhibition occurs in cellular or tissue systems. Highly active PFK with altered intracellular distribution and regulation is prevalent in tumor tissues.^38^, ^39^, ^40^, ^41^

**Figure 2:**
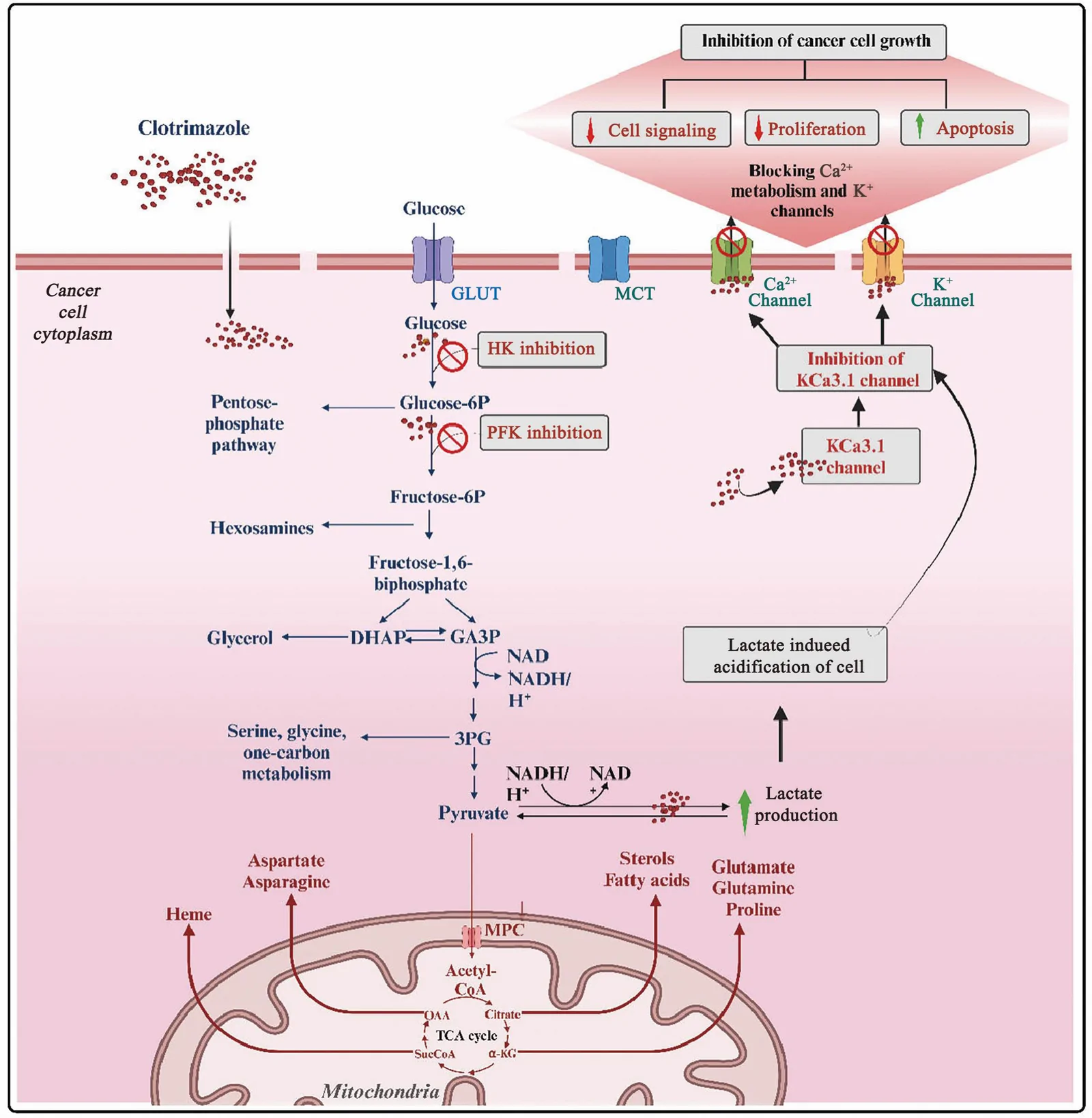
Anticancer mechanism of clotrimazole. 3PG: 3-Phosphoglycerate; 6P: 6-Phosphate; α-KG: Alpha-ketoglutarate; Ca^2+^: Calcium ion; Acetyl-CoA: Acetyl-coenzyme A; DHAP: Dihydroxyacetone phosphate; GA3P: Glyceraldehyde-3-phosphate; GLUT: Glucose transporter; H^+^: Hydrogen ion; HK: Hexokinase; K^+^: Potassium ion; KCa3.1: Intermediate conductance calcium-activated potassium channel 3.1; MCT: Monocarboxylate transporter; MPC: Mitochondrial pyruvate carrier; NAD: Nicotinamide adenine dinucleotide; NADH: Reduced nicotinamide adenine dinucleotide; OAA: Oxaloacetate; PFK: 6-Phosphofructo-1-kinase; SucCoA: Succinyl-coenzyme A; TCA cycle: Tricarboxylic acid cycle.

CLZ also has the ability to cause G1-phase arrest, and compared with healthy cells, malignant cells require more energy to proliferate.^42^ In the rate-limiting stages of glycolysis, hexokinase (HK), a glycolytic enzyme, is crucial. The four isoforms of HK (HK1, HK2, HK3, and HK4) are highly expressed on the surface of cancer cells and cover the outermost layer of mitochondria via transmembrane voltage-dependent anion channels.^43^^,^^44^ By blocking HK glycolytic enzymes, CLZ up-regulate cancer apoptotic factors and induce cell death. Moreover, aldolase and *PFK*, another glycolytic enzyme, are inhibited by CLZ. Tumor cells can multiply or behave aggressively; however, CLZ may stop this. Blocking Ca^2+^-activated K^+^ channels and restricting Ca^2+^ metabolism are other primary mechanism of CLZ activity. These inhibit the growth of malignant cells, as shown in Figure 2.^7^ CLZ has been shown to be highly effective against MDA-MB-231 and other invasive breast cancer cell lines. It functions by causing apoptosis, G1-phase cell cycle arrest, and glycolytic inhibition. Particularly, in MDA-MB-231 cells, CLZ inhibits glycolytic enzymes and lowers glucose absorption, leading to a reduction in adenosine triphosphate (ATP) production.^38^ Moreover, it disrupts proliferation and accelerates cell death by inducing G1-phase arrest, boosting pro-apoptotic proteins, and lowering anti-apoptotic signals.^45^ Less aggressive cancer cell lines, including MCF-7, show less response, indicating stronger sensitivity to aggressive tumors, with a considerable decline in viability and migration.^38^

In addition to breast cancer, CLZ has proven anticancer promise against numerous other malignancies, including glioblastoma and colorectal, lung, and melanoma cancers. Studies have shown that CLZ interferes with mitochondrial activity, leading to oxidative stress and death in glioblastoma cells. In lung and colorectal cancer models, CLZ has been found to affect important oncogenic pathways such as *PI3K/*protein kinase B (*Akt*) and adenosine monophosphate-activated protein kinase (*AMPK*)*/*mammalian target of rapamycin (*mTOR*), leading to tumor suppression. The ability of CLZ to target several metabolic and signaling pathways makes it an attractive option for combination therapy, perhaps increasing the efficacy of current chemotherapeutics or targeted treatments.^46^ Further preclinical and clinical studies are needed to examine CLZ's full therapeutic potential, improve its dose regimens, and analyze its safety profile in patients with cancer.

*In vitro* studies on breast cancer have shown that CLZ inhibits glycolysis by blocking PFK activation and suppressing other glycolytic enzymes, resulting in decreased ATP generation and increased oxidative stress. Furthermore, CLZ induces apoptosis, inhibits cancer cell invasion, and inhibits matrix metalloproteinase-9, indicating its potential for repurposing for cancer treatment. Similar findings in liver cancer suggest that CLZ decreases metastasis by altering extracellular signal-regulated kinase (ERK)*-*p65 signaling and slowing epithelial-mesenchymal transition (EMT). Research findings in prostate and colon cancers demonstrate that CLZ increases the effectiveness of the CPP2 peptide, displaying synergistic benefits when paired with specific pharmacological changes.^38^^,^^45^^,^^47^

In melanoma, both *in vitro* and *in vivo* studies have shown that CLZ reduces ATP levels, glycolysis, and tumor development while suppressing tumor-associated macrophages (TAMs). CLZ also inhibits *HK-*mitochondrial connections, causes apoptosis, and increases sensitivity to chemotherapy. CLZ inhibits Ca^2+^-activated K^+^ channels in endometrial cancer, slowing tumor growth in both cell and animal models. These results collectively indicate that CLZ, an antifungal drug, exerts strong anticancer effects via various pathways, making it an attractive candidate for drug repurposing in oncology.^8^^,^^43^^,^^48^

## Molecular targets and signaling pathways

Although the exact mechanisms of action of CLZ in cancer treatment are still being investigated, research indicates that CLZ may affect several cellular processes related to carcinogenesis and cancer progression.

### Targeted protein and receptor

According to several studies, CLZ's anticancer activities include interactions with several proteins and receptors linked to the initiation and spread of cancer. According to these studies, CLZ affects Ca^2+^ and K^+^ ion channels, which are crucial for apoptosis, migration, and cell division.^49^ CLZ can induce apoptosis in cancer cells by blocking ion channels and disrupting intracellular Ca^2+^ homeostasis. CYP450 enzymes, which are involved in the metabolism of both endogenous substances and xenobiotics, are also inhibited by CLZ. K^+^ and Ca^2+^ blockers' effects and their mode of action on metastatic melanoma cell lines are being investigated. CLZ's alteration of CYP450 activity may affect the metabolism of endogenous substrates that are engaged in cell signaling pathways or the transformation of prodrugs into cytotoxic agents in cancer cells.^3^^,^^50^

CLZ also blocks critical glycolytic enzymes, such as *PFK* and *HK*, which are crucial for cancer cell energy metabolism. By interrupting glycolysis, CLZ promotes metabolic stress and energy depletion in tumor cells, resulting in growth suppression. Furthermore, CLZ interacts with mitochondrial permeability transition pores, causing mitochondrial malfunction and initiating intrinsic apoptosis. It also affects membrane-bound receptors such as epidermal growth factor receptor (EGFR) and insulin-like growth factor receptor, which are typically overexpressed in aggressive tumors. These interactions indicate that CLZ's mechanism of action extends beyond ion channel blockade, making it a versatile anticancer drug with potential implications in combination treatment.^51^

### Intracellular signaling cascades

Previous studies have suggested that various of antifungal drugs affect the intracellular signaling cascades connected to cancer pathogenesis. CLZ inhibits the Wnt/β-catenin signaling system, which controls stem cell maintenance, differentiation, and proliferation.^52^ β-catenin is reduced by CLZ in a proteasome-independent manner. Through the RNA-induced protein kinase and heme-regulated translational inhibitor genes being knocked down, Yonezawa et al.^52^ showed that these translational regulators mediate the CLZ-induced suppression of the Wnt/β-catenin pathway. Therefore, by reducing the amounts of β-catenin protein via translational control, CLZ suppresses the Wnt/β-catenin pathway. Several types of cancers exhibit dysregulation of this pathway, and CLZ-mediated inhibition decreases the proliferation of cancer cells while promoting cell differentiation.^53^ CLZ has the potential to alter the signaling pathway of mitogen-activated protein kinase (MAPK), including the ERK pathway, which governs cell survival, proliferation, and differentiation. According to a study on hepatocellular carcinoma (HCC) by Li et al.*,*^54^^,^^55^ CLZ decreases EMT by altering the *ERK* dephosphorylation-dependent pathway, which further inhibits the proliferation, migration, and invasion of HCC. The disruption of MAPK/ERK signaling suppresses the growth of cancer cells and triggers cell death.

Apart from Wnt/β-catenin and MAPK/ERK pathways, CLZ has been demonstrated to affect the PI3K/Akt/mTOR signaling cascade, which plays a vital role in cancer cell survival and treatment resistance. By decreasing Akt phosphorylation, CLZ affects downstream signaling, resulting in the inhibition of cell growth and induction of apoptosis. Moreover, CLZ affects the Janus kinase (JAK)/signal transducer and activator of transcription (STAT) pathway, which is a major regulator of immune responses and tumor growth. Reducing STAT3 phosphorylation reduces the production of anti-apoptotic proteins, including Bcl-2 and survivin, making cancer cells more vulnerable to programmed cell death.^56^ These findings imply that CLZ's anticancer efficacy is related to its capacity to interfere with various intracellular signaling cascades, highlighting its potential as a repurposed anticancer drug.

### Immunomodulatory effects

The field of immunomodulation is gaining significant attention owing to its potential for use in fundamental research and treatment.^57^ The anticancer effects of CLZ also involve immunomodulatory pathways, such as inflammatory mediators and immunological checkpoint regulators, which affect the tumor microenvironment (TME) and immune responses against cancer. CLZ regulates the synthesis of inflammatory mediators, including prostaglandins and cytokines, which can affect immune cell activity and tumor-related inflammation. CLZ has the potential to improve antitumor immune responses and prevent cancer progression by altering the inflammatory state inside the TME.^58^ According to a study by Thapa et al.,^59^ pro-inflammatory cytokine interleukin (IL)-8 production is downregulated, and the epithelial and endothelial responses to IL-8 are blocked, causing the suppression of inflammatory angiogenesis.

Another benefit of CLZ is that it alters immunological checkpoint pathways that are critical for immune evasion by cancer cells. By downregulating the expression of Programmed Cell Death Ligand 1, a critical immunological checkpoint protein, CLZ may improve T-cell-mediated immune responses against malignancies. Furthermore, CLZ affects macrophage polarization by moving TAMs from the pro-tumorigenic M2 phenotype to the antitumorigenic M1 phenotype, thereby creating a more immunogenic TME. This change can lead to increased release of pro-inflammatory cytokines like tumor necrosis factor-alpha (TNF-α) and interferon-gamma (IFN-γ), further boosting the immune system's capacity to attack cancer cells. These findings indicate that CLZ's immunomodulatory actions may complement existing immunotherapies, making it a suitable option for combination therapy in cancer treatment.^60^

## Combination therapies and novel drug delivery systems for clotrimazole

The therapeutic effectiveness of CLZ as an anticancer treatment can be improved using combination therapy and novel drug delivery systems. CLZ can be used in combination with other chemotherapeutic drugs to target several pathways involved in cancer development. This can help reduce drug resistance and enhance treatment effectiveness. Sharma et al.^61^ combined CLZ with conventional chemotherapeutic agents such as paclitaxel (PAX). The combination of PAX and CLZ affected breast cancer cells by increasing oxidative and nitrogen stress, causing genotoxicity, and decreasing glucose uptake, with minimal effects on normal cells. Moreover, a more personalized approach to cancer treatment may be possible by combining CLZ with medicines that target certain biological targets linked to cancer development, such as angiogenesis inhibitors and receptor tyrosine kinases.^6^ According to Sung et al.,^62^ CLZ and ketoconazole in combination have a strong anticancer action in breast cancer cells by preventing cell division, triggering apoptosis, and causing G1-phase cell cycle arrest. This combination also inhibits matrix metalloproteinase, which reduces the invasiveness of MDA-MB-231 cells.^45^ A formulation of novel combinations based on metals, such as ruthenium (Ru) with CLZ, was carried out by Elisa et al.^63^ According to this study, the combination exhibited decreased cytotoxicity against non-transformed epidermal cell lines, but increased cytotoxicity against prostate cell lines. Tarek et al.^64^ showed that imatinib combined with CLZ improved the ability of T47D breast cancer cells to limit cell proliferation. By integrating CLZ into novel drug delivery devices, we can improve its pharmacokinetic–pharmacodynamic profile and tumor-targeting abilities. Drug delivery technologies based on nanoparticles, including lipid or polymeric nanoparticles, can specifically facilitate the delivery of CLZ to tumor tissues while reducing systemic toxicity. Through the increased permeability and retention (EPR) effect, these nanocarriers can passively accumulate in tumors, enabling the prolonged release of CLZ inside the TME. Numerous studies have been conducted on the applicability of CLZ which also includes the formulation of CLZ in nano micellar form, chitosan nanoparticles,^65^ self-assembling nano-micelles of glycyl-glycine analogs of amphiphilic CLZ,^48^ multilayer films based on chitosan/pectin polyelectrolyte complexes,^66^ cross-linkable star-hyperbranched unimolecular micelles,^67^ compounds of platinum-CLZ,^68^ palladium-CLZ combination.^69^ to explore the potential of CLZ for ferroptosis activation, targeting cancer stem cells, and epigenetic modulation by influencing histone changes and deoxyribonucleic acid methylation. It may also disrupt cancer-associated fibroblasts (CAFs), interfere with hypoxia-inducible factor-1 alpha signaling, and increase drug penetration in malignancies. Incorporating proteolysis targeting chimera technology or bioconjugates with antibodies or peptides may boost CLZ specificity and effectiveness. Three-dimensional tumor models and organoids can be used to optimize CLZ-based therapy, making it a viable agent in modern oncology. These studies demonstrated the improved anticancer activity of CLZ-loaded and complex formulations. Moreover, enhancing tumor selectivity and drug release kinetics by modifying the surface of nanoparticles with stimuli-responsive moieties or targeting ligands may improve treatment effectiveness.

## Other pharmacological activities of clotrimazole

### Cardiovascular effects

Although CLZ is not directly linked to cardiovascular effects, recent studies have suggested possible cardioprotective benefits of this drug. According to a study by Tian et al.*,*^70^ CLZ strongly suppresses human cardiac repolarization K^+^ currents, such as Ito1, IKur, IhERG, and IKs, in isolated cells in a concentration-dependent manner. CLZ affects ion channels related to cardiac electrophysiology, including Ca^2+^ and K^+^ channels, which are essential for the heart's excitation-contraction coupling. CLZ's vasodilatory effects have been studied, possibly due to the relaxation of smooth muscle cells in the vascular system. Although further investigations are required to explain the specific cardiovascular effects and therapeutic consequences of CLZ, these results suggest that the drug may be useful for treating cardiovascular diseases characterized by irregular ion channel activity or vascular dysfunction.^71^ CLZ may have cardiovascular effects, including vasodilation and blood pressure reduction, and possibly cardioprotection. It blocks K^+^ channels, raising concerns regarding QT prolongation and arrhythmia risk. CLZ may potentially boost endothelial function, lower the risk of atherosclerosis, and minimize chemotherapy-induced cardiotoxicity. However, further studies are required to validate its therapeutic potential in cardiovascular disorders.

### Anti-inflammatory properties

In addition to its antifungal effects, CLZ also has anti-inflammatory properties. Evidence suggests that it suppresses the synthesis of pro-inflammatory cytokines, including TNF-α and IL-6, and regulates the activity of inflammatory enzymes, such as lipoxygenase (LOX) and cyclooxygenase (COX).^72^^,^^73^ Further research is required to confirm CLZ's safety and effectiveness in these indications. CLZ's anti-inflammatory properties suggest possible uses in the management of inflammatory diseases, such as rheumatoid arthritis and inflammatory bowel disease. CLZ exhibits anti-inflammatory properties by decreasing nuclear factor-kappa B (NF-κB) signaling, reducing oxidative stress, and altering macrophage polarization. It downregulates the COX-2 and LOX pathways, lowering prostaglandin and leukotriene synthesis, respectively. CLZ may decrease inflammasome activation, lowering IL-1β release and inflammation-associated tissue damage. Its possible functions in neuroinflammation, chronic inflammatory diseases, and cancer-related inflammation warrant further investigation.^74^

### Neuroprotective potential

CLZ is a potential alternative treatment for neurological illnesses because of its neuroprotective properties. It alters the activity of neuronal ion channels, such as Ca^2+^ and K^+^ channels, which are essential for neurotransmitter release and neuronal excitability. Its capacity to reduce oxidative stress and neuronal apoptosis has been studied for protection against neurodegenerative processes.^53^ CLZ's neuroprotective benefits extend to suppressing glutamate excitotoxicity, decreasing neuroinflammation, and boosting mitochondrial function. It controls Ca^2+^ homeostasis and prevents neuronal hyperexcitability associated with neurodegenerative illnesses, including Alzheimer's and Parkinson's diseases. CLZ's antioxidant qualities help counteract oxidative stress, which plays a critical role in neuronal damage. Its potential for treating epilepsy, multiple sclerosis, and ischemic stroke is currently being examined, indicating its flexibility beyond antifungal treatments.^53^

### Immunomodulation

The immunomodulatory effects of CLZ may affect several immune-related conditions. CLZ has been shown to regulate immune cell activity, including lymphocyte and macrophage functions, as well as cytokine and chemokine synthesis, which are involved in immunological responses.^75^ The effects of CLZ on immunological checkpoint molecules, including cytotoxic T-lymphocyte-associated protein 4 (CTLA-4) and programmed cell death protein 1 (PD-1), which are important regulators of T-cell activation and immune tolerance, have been studied.^76^ Its immunomodulatory effects point to possible uses of the drug in the treatment of autoimmune illnesses, organ transplant rejection, or cancer immunotherapy. CLZ's immunomodulatory effects extend to lowering chronic inflammation and adaptive immune responses. It has shown potential for lowering hyperactive immune responses in autoimmune illnesses, including lupus and rheumatoid arthritis. Thus, CLZ may boost the effectiveness of immune checkpoint inhibitors in cancer treatment by modifying T-cell exhaustion and increasing antitumor immunity. Its ability to downregulate pro-inflammatory mediators while increasing regulatory T-cell activity suggests its potential use in graft-versus-host and inflammatory diseases. Further studies are required to evaluate its clinical feasibility in immunotherapy.^53^

## Future perspectives

CLZ has significant potential when used in combination with chemotherapy, immunotherapy, or targeted medicines to enhance outcomes and overcome drug resistance. Although CLZ has potential as an anticancer drug, numerous crucial areas require further exploration to achieve clinical success. Key factors include completing extensive clinical studies because sufficient information is not available for CLZ to be used as an anticancer drug, and to confirm its safety and effectiveness. Nanotechnology-based formulations, such as nanoparticles and hydrogels, could enhance CLZ's absorption and tumor targeting while reducing toxicity. Understanding CLZ's molecular mechanisms, including its effects on glycolysis, ion channels, and signaling cascades, is crucial. Utilizing omics approaches may provide deeper insight and help identify predictive biomarkers.

Emerging research on CLZ's immunomodulatory properties, particularly its interactions with *PD-1* and *CTLA4*, has highlighted its potential role in cancer immunotherapy. In addition to its oncological applications, exploring its cardiovascular, neuroprotective, and anti-inflammatory properties may lead to new therapeutic applications. Solving pharmacokinetic challenges such as low solubility and systemic toxicity through molecular modifications or advanced formulations is essential. Future research should prioritize overcoming these limitations to realize CLZ's potential as a safe, effective, and versatile cancer treatment.

Despite its comprehensive approach, this study had a few limitations. This review is based on current data, which does not cover all the relevant information on CLZ's anticancer activity. There is a lack of precise clinical data in this review, and additional studies are necessary to establish its efficacy. To address these limitations and offer greater knowledge on the potential of CLZ in cancer treatment, future research demands more emphasis on clinical trials and novel delivery approaches.

## Conclusions

This review highlights the recent advancements in our understanding of CLZ's role in inhibiting carcinogenesis and metastasis in various cancer types. Although traditionally used as an antifungal agent, it has been found to exhibit anticancer properties, positioning CLZ as a promising anticancer agent. However, current research fails to fully assess the anticancer potential of CLZ, and more detailed mechanistic and systematic *in vivo* studies are needed to enhance our understanding and support its clinical development. CLZ has shown significant bioactivity in both *in vitro* and *in vivo* studies across multiple types of cancer, with the strongest effects observed in breast cancer and melanoma [Table 1].

**Table 1:** Summary of CLZ anticancer activity across different cancer types: study models, mechanisms, effects, and key tests.

Cancer type	Study model	Mechanism	Experimental methods	Key outcomes	Reference
Breast cancer	*In vitro*	Reduction in the particulate matter linked to *PFK* activation.	*PFK* activity; f-actin and *PFK* sedimentation; glucose uptake and lactate generation.	In human breast tumor tissues, CLZ interferes with glycolysis, which prevents *PFK* from acting by separating the enzyme from f-actin.	Coelho et al.^39^
	*In vitro*	Suppressing the activity of other cytosolic, mitochondrial, and glycolytic enzymes. There was an increase in the synthesis of lactate, intracellular ATP, reactive oxygen species (ROS), and antioxidant potential.	Redox potential; 3- (4,5-dimethylthiazol-2-yl)-2,5-diphenyltetrazolium bromide assay, quantitative measurement of ATP; activity of succinate dehydrogenase; Giemsa-stained optical microscopy; evaluation of glycolytic enzyme levels	Compared with MCF-7 cells, CLZ responds more sensibly to MDA-MB-231 cells. This was particularly noticeable when it came to antioxidant capacity, which is a crucial cell defense against drugs that alter cell metabolism. Also, CLZ's potential utility as an anticancer drug is strengthened by its water-soluble formulation.	Marcondes et al.^47^
	*In vitro* and *in vivo*	Apoptotic activation, G1 arrest, and invasiveness control by inhibition of matrix metalloproteinase-9	Xenograft tumor tissue fractionation; protein extraction and immunoblot analysis; flow cytometry; assessment for apoptosis.	Because of their well-established pharmacokinetic profiles and toxicity, imidazole antifungal drugs have the potential to be drug repurposed in anticancer therapies. The article's findings show that these generic drugs have potent antitumor effects on breast cancer cells.	Bae et al.^77^
	*In vitro*	Inhibits glycolytic flow by interfering with *PFK* and F-actin co-localization.	SEM, cell fractionation, glucose intake and lactate generation, cytotoxicity assay, enzyme activity measurement, assays for cytochemistry and immunocytochemistry	These results confirm that CLZ causes apoptosis in MCF-7 cells by inhibiting, glycolytic flow, indicating that it may be a viable treatment for breast cancer in humans.	Meira et al.^45^
	*In vitro*	Direct *HK* inhibition	Assays for cell viability, absorption of glucose, quantification of mitochondrial activity and ATP; enzyme activity; migration; and proliferation assay	The drug inhibits pyruvate kinase, *PFK*, *HK*, and other important glycolytic enzymes within cells and lowers the amount of ATP present within them, particularly in the highly metastatic breast cancer cell line (MDA-MB-231)	Furtado et al.^38^
Liver cancer	*In vitro*	Inhibits migration and invasion of HCC by altering *ERK*-*p65* signaling.	Western blotting, migration and invasion, wound healing, cell growth assay	The results of this research suggest that CLZ prevents HCC metastasis by inhibiting EMT in a way that is based on *ERK* dephosphorylation.	Liu et al.^55^
Prostate and colon cancer	*In vitro*	Not specified	Assays for cytotoxicity, drug interaction analysis, synergistic assessment	The anticancer efficaciousness of CPP2 peptide was reported to be enhanced in PC-3 and HT-29 cells with modification of its N-terminal. When CPP2-thiazole conjugates were coupled with CLZ instead of PTX, PC-3 cells exhibited increased sensitivity and more synergistic effects.	Duarte and Vale^78^
Melanoma	*In vitro*	Efficiently decrease the amount of ATP and glycolysis in B16 melanoma cells.	Measuring proteins, separating and testing soluble and mitochondrial *HK*	Because CLZ and bifonazole separate glycolytic enzymes from the cytoskeleton, they efficiently lower ATP levels and glycolysis in B16 melanoma cells. Furthermore, they cause *HK* to separate from mitochondria in a dose-dependent manner.	Penso and Beitner^43^
	*In vitro* and *in vivo*	*PI3K* inhibitor both *in vitro* and *in vivo*, as well as causing tumor-associated macrophages to repolarize.	Western blotting, qPCR, the cell viability test, the intake of glucose and the generation of lactate, and mitochondrial reductive activity assays for polarization of macrophages obtained from bone marrow and mice.	In a mouse model of implanted melanoma, CLZ suppressed tumor development, lowered the amount of lactate in the TME, and lowered the expression of vascular endothelial growth factor. Lastly, CLZ significantly reduced the tumors' TAM invasion, which caused M1 polarization. All of these results point to a novel way that CLZ fights cancer by influencing the TME, namely the viability and activity of TAM.	Ochioni et al.^8^
	*In vitro* and *in vivo*	Blocks G1 phase arrest, Ca2^+^-dependent K^+^ channels, and the glycolytic enzymes *PFK*, aldolase, and *HK*.	Assays for measuring hydrogen peroxide, reduced and total glutathione concentration, *in vitro* apoptosis, cell proliferation, and Western blotting.	Self-assembling CLZ glycyl glycine nano micelles, or CLT-GG-SANMs, improves melanoma cell death and drug delivery. Both *in vitro* and *in vivo*, they demonstrated higher therapeutic efficiency in triggering apoptosis and tumor regression due to their tiny size and efficient drug release. CLT-GG-SANMs have potential as a melanoma therapy that is clinically feasible.	Kaur et al.^48^
	*In vitro*	*HK* type-II expression is decreased by CLZ, which also causes DNA fragmentation without necrosis, annexin V reactivity to change, cell cycle arrest at the G1-S phase transition, and cell cycle arrest.	Gene expression, cell cycle progression, fragmentation of internucleosomal DNA, and annexin V reactivity.	According to the study, CLZ and cisplatin both have pro-apoptotic effects on human melanoma cells. Also, CLZ modifies the glycolysis pathway and causes cell cycle arrest at the G1-S phase transition, which may make tumor cells more susceptible to chemotherapy and radiation.	Adinolfi et al.^79^
Endometrial cancer	*In vitro* and *in vivo*	Blockage of Ca^2+^ -activated K^+^ channels with moderate conductance.	Western blotting, reverse transcription, real-time polymerase chain reaction, cell cycle analysis, incorporation of [3H] thymidine, and electrophysiology.	CLZ, TRAM-34, charybdotoxin, and siRNA targeting IKCa1 all dramatically decreased the CLZ-sensitive K^+^ current that was produced in EC cells by elevated Ca^2+^. When EC cells were introduced into nude mice, TRAM-34 and CLZ inhibited the growth of tumors. The formation of EC requires increased IKCa1 channel activation.	Wang et al.^80^

3H-thymidine: Radioactively labeled thymidine; ATP: Adenosine triphosphate; B16: B16 murine melanoma cells; Ca^2+^: Calcium; CLZ: Clotrimazole; CPP2: Casein kinase-2 inhibitor; EC: Endometrial cancer; EMT: Epithelial-mesenchymal transition; ERK: Extracellular signal-regulated kinase; F-actin: Filamentous actin; G1: Gap 1 phase of the cell cycle; G1-S: Transition from gap 1 to synthesis phase in the cell cycle; HCC: Hepatocellular carcinoma; HK: Hexokinase; HT-29: Human colorectal adenocarcinoma cell line; IKCa1: Intermediate conductance calcium-activated potassium channel; K^+^: Potassium; M1: M1 macrophage phenotype; MCF-7: Michigan Cancer Foundation-7 human breast cancer cell line; MDA-MB-231: Human triple-negative breast cancer cell line; MTT assay: 3-(4,5-dimethylthiazol-2-yl)-2,5-diphenyltetrazolium bromide assay; p65: NF-κB subunit; PC-3: Prostate cancer cell line-3; PFK: Phosphofructokinase; PI3K: Phosphatidylinositol 3-kinase; PTX: Paclitaxel; qPCR: Quantitative polymerase chain reaction; ROS: Reactive oxygen species; SEM: Scanning electron microscopy; siRNA: Small interfering RNA; TAM: Tumor-associated macrophages; TME: Tumor microenvironment; TRAM-34: Triarylmethane-34.

CLZ's anticancer activity involves several critical signaling pathways, including the glycolytic, *ERK*-*p65*, *PI3K*, and mitochondrial pathways. *In vitro* studies in various cell lines have shown that CLZ induces apoptosis and modulates oncogenic pathways. Specifically, in breast cancer, CLZ affects glycolytic enzymes, such as PFK and HK, leading to reduced ATP production and increased apoptosis. In HCC, CLZ disrupts extracellular signal-regulated kinase-NF-κB subunit; (ERK-p65) signaling and inhibits cell migration and invasion by preventing EMT. In melanoma, CLZ inhibits glycolytic enzymes, causes the dissociation of *HK* from the mitochondria, and affects the *PI3K* pathway, resulting in the repolarization of TAMs and reduced tumor growth. In endometrial cancer, CLZ blocks Ca^2+^-activated K^+^ channels, particularly IKCa1, which results in decreased cell proliferation. These mechanisms demonstrate CLZ's therapeutic potential as a repurposed drug capable of disrupting various aspects of cancer metabolism, migration, and apoptosis. While *in vivo* studies using xenograft mouse models have shown that CLZ promotes apoptosis and inhibits *MMP-9* in breast cancer, repolarizes macrophages, disrupts glycolysis in melanoma, and blocks Ca^2+^ -activated K^+^ channels in endometrial cancer, further research is needed.

Currently, there are only a few studies on the therapeutic dose and toxicity of CLZ in humans. Further development of bioavailability assays, dose–response curves, and molecular modifications of CLZ's chemical structure are needed to gain a deeper understanding of its pharmacokinetics and pharmacodynamics. Given these encouraging results, CLZ appears to be a promising multi-target agent for cancer prevention and treatment.

## Authors contribution

Shubham C Karpe and Manjula Kiran conceived the study and developed the theory; Shubham C Karpe, Shukhen Manjhi, and Jaipal Meena performed the data curation; Manjula Kiran, Rajesh Kumar, and Harish Chander verified the data and supervised the findings of this work, along with Anupkumar R. Anvikar and Harit kasana. All authors discussed the results and contributed to the final manuscript.

## Ethics statement

None.

## Declaration of generative AI and AI-assisted technologies in the writing process

The authors declare that generative artificial intelligence (AI) and AI assisted technologies were not used in the writing process or any other process during the preparation of this manuscript.

## Funding

None.

## Data availability statement

The datasets used in the current study are available from the corresponding author on reasonable request.

## Conflict of interest

The authors declare that they have no known competing financial interests or personal relationships that could have appeared to influence the work reported in this paper.
